# Specific Behavioral Responses Rather Than Autonomic Responses Can Indicate and Quantify Acute Pain among Individuals with Intellectual and Developmental Disabilities

**DOI:** 10.3390/brainsci11020253

**Published:** 2021-02-18

**Authors:** Ruth Defrin, Tali Benromano, Chaim G. Pick

**Affiliations:** 1 Department of Physical Therapy, School of Health Professions, Sackler Faculty of Medicine, Tel-Aviv University, Tel Aviv 69978, Israel; 2 Sagol School of Neuroscience, Tel-Aviv University, Tel Aviv 69978, Israel; pickc@tauex.tau.ac.il; 3 Department of Anatomy, Sackler Faculty of Medicine, Tel-Aviv University, Tel Aviv 69978, Israel; talibenromano@gmail.com; 4 Miriam and Sheldon G. Adelson Chair and Center for the Biology of Addictive Diseases, Tel-Aviv University, Tel-Aviv 69978, Israel; 5 Sylvan Adams Sports Institute, Tel Aviv University, Tel Aviv 69978, Israel

**Keywords:** cognitive impairment, intellectual disability, experimental pain, pain measurement, facial action, self-report, autonomic responses

## Abstract

Individuals with intellectual and developmental disabilities (IDD) are at a high risk of experiencing pain. Pain management requires assessment, a challenging mission considering the impaired communication skills in IDD. We analyzed subjective and objective responses following calibrated experimental stimuli to determine whether they can differentiate between painful and non-painful states, and adequately quantify pain among individuals with IDD. Eighteen adults with IDD and 21 healthy controls (HC) received experimental pressure stimuli (innocuous, mildly noxious, and moderately noxious). Facial expressions (analyzed with the Facial Action Coding System (FACS)) and autonomic function (heart rate, heart rate variability (HRV), pulse, and galvanic skin response (GSR)) were continuously monitored, and self-reports using a pyramid and a numeric scale were obtained. Significant stimulus-response relationships were observed for the FACS and pyramid scores (but not for the numeric scores), and specific action units could differentiate between the noxious levels among the IDD group. FACS scores of the IDD group were higher and steeper than those of HC. HRV was overall lower among the IDD group, and GSR increased during noxious stimulation in both groups. In conclusion, the facial expressions and self-reports seem to reliably detect and quantify pain among individuals with mild-moderate IDD; their enhanced responses may indicate increased pain sensitivity that requires careful clinical consideration.

## 1. Introduction

Intellectual disabilities are defined by the Diagnostic and Statistical Manual of Mental Disorders (DSM, 5th Edition) as neurodevelopmental disorders that begin in childhood and are characterized by intellectual difficulties as well as difficulties in conceptual, social, and practical areas of living. These include confirmed deficits in reasoning, problem solving, planning, judgment, and learning from experience, among others, as well as deficits in adaptive functioning and independence. Individuals with intellectual and developmental disabilities (IDD) are at an increased risk of experiencing pain than are the cognitively intact population [1] for several reasons. First, the etiology of the IDD and its subsequent complications may affect multiple bodily systems; it may also require painful diagnostic tests and procedures [2,3,4,5]. Second, individuals with IDD exhibit greater rates than normal of falls, accidents, and injuries [6,7]. Third, individuals with IDD may be limited in their ability to comprehend the implications of an injury or pain and to adequately communicate it to care givers; thus, they may not receive a proper diagnosis or care at a proper timing [8,9,10]. Finally, the overall low level of physical activity [11] and the reduced involvement in health decision making [12] may further increase the risk for pain in individuals with IDD. 

The unfortunate consequence of increased exposure to many potentially painful situations, along with the limited cognitive and communication abilities of individuals with IDD, is that these individuals seem to receive less treatment for pain than their cognitively intact peers [8,13,14]. Furthermore, the prevalence of chronic pain among individuals with IDD, reaching up to 70%, is high by any standard [13,15,16]. Identifying and quantifying pain states among individuals with IDD in a manner that does not necessitate self-reporting is thus crucial in order to provide them with adequate pain management; however, it is also particularly challenging (for a review, see reference [17]). 

Behavioral and physiological indices are potential candidates for this purpose. Indeed, individuals with IDD respond to painful clinical conditions, such as dental care, venipuncture, or physical treatment, with increased facial and bodily expressions, e.g., [18,19,20,21,22,23,24] as well as increased heartbeat, blood pressure, and oxygen saturation [25,26,27], compared with baseline values. However, because pain-provoking stimuli in these conditions can neither be quantified nor controlled, the extent to which such behavioral and physiological indicators can reliably differentiate between non-painful and painful states, and between different levels of pain, requires additional investigation and verification.

In two pioneering studies, innocuous light touch, deep pressure, cool and warm stimuli, and pin prick induced similar increases in facial-body reactions compared to sham trials among adults with severe-profound IDD [28,29]. However, comparisons to normative responses were unavailable. Similar stimuli among children with IDD induced overall increased facial and body responses, compared with control children [30]; however, there was appreciable inter- and intra-individual variability [31]. Therefore, differentiating pain responses from responses to innocuous stimuli was very challenging. In another study, individuals with IDD and controls could similarly discriminate between sharp and dull pinpricks; however, the sharp stimulus was not necessarily painful [32]. 

Previously, we measured the behavioral and autonomic responses of individuals with cerebral palsy (CP) and IDD vs. cognitively intact controls to standardized innocuous and noxious mechanical stimuli. We found a gradual increase in the magnitude of the facial expressions (but not of the autonomic signs) with an increase in stimulation intensity, which was steeper among the IDD group [33]. However, it is not clear whether these responses were characteristic of CP and were affected by the motor impairment of the participants, or if they were typical of IDD in general; this requires further investigation. In a later study, we found greater pain-evoked potentials among individuals with IDD than in controls, which was correlated with stimulation intensity [34]; however, this assessment method may not be feasible for clinical purposes. Except for another study in which tactile stimuli produced an increase in facial temperature among children with IDD, compared with controls [24], we could not find other studies in which physiological indices have been analyzed following experimental noxious stimuli. 

Owing to the dearth of studies assessing pain responses to standardized noxious stimuli among individuals with IDD, and considering the possibility that these individuals may be pain hypersensitive [35,36], our aim was to determine whether objective, semi-objective, and subjective pain indicators among individuals with IDD following standardized stimuli: (1) can differentiate between pain and non-pain states, (2) can reliably indicate pain intensity, and (3) are increased compared with controls.

## 2. Materials and Methods

### 2.1. Participants

The study comprised 39 adults: 18 individuals with IDD (IDD group, age 36.4 ± 7.5 years) and 21 cognitively intact healthy controls (HC group, 32.4 ± 9.2 years). All the individuals with IDD were recruited from a central daycare center for people with IDD (Israel Elwyn). IDD was diagnosed based on clinical evaluation and standardized testing of intelligence (including the Wechsler Intelligence Scale for Children-Revised and the Wechsler Preschool and Primary Scale of Intelligence), performed by a team from the national Ministry of Social Affairs and Social Services, which supervises all services related to IDD. These individuals had an estimated level of mild or moderate IDD, and the ability to understand their mother tongue. Healthy controls were employees or students of Tel-Aviv University or of the day-care center for people with IDD. Exclusion criteria were as follows: any known acute or chronic pain (all the participants), bruises or injuries in the testing regions (all the participants), and idiosyncratic behaviors such as self-injury and moaning (the individuals with IDD). Medical information on the participants with IDD was retrieved from their medical records by their legal guardian upon request, and additional information was also obtained from the primary care giver and the day care center physician if needed. The study was conducted in accordance with the Declaration of Helsinki, and the protocol was approved by the Ethics Committee of Tel-Aviv University (3012/2012), the institutional review board of the Ministry of Social Affairs and Social Services (201323-01), and by the legal guardians of the participants with IDD. Prior to entering the study, a written informed consent was attained from all the participants of the control group and from the legal guardians of all the participants with IDD, after they received explanations on the study’s aims and protocols. In addition, the protocol was explained to the participants with IDD and their escorts upon their arrival to the lab, and each step of the protocol was carried out only after their oral consent was obtained.

### 2.2. Instruments

#### 2.2.1. Pressure Algometer

Pressure stimuli were delivered, using a pressure algometer (Somedic Sales AB, Algometer type II, Sweden). The algometer has a pressure transducer unit, an electronic recording and display unit, and a subject-activated push button connected via a cable to the algometer. Its accuracy level is ±3% and its unit of measurement is the kilopascal (kPa). The examiner holds the algometer perpendicular to the skin surface and applies a constantly increasing pressure (with pre-determined rates) that is monitored and viewed on the screen of the electronic display. The surface area of the algometer’s probe that is pressed against the skin was 1 cm^2^. 

#### 2.2.2. PMD-100 System 

Autonomic responses to pressure stimuli were evaluated by measuring the fluctuations in the heart rate (HR), heart rate variability (HRV), photoplethysmograph wave amplitudes (PPGA), and galvanic skin response (GSR). These physiological signals were constantly recorded, sampled, and stored on a computer using the PMD-100 system (Medasense Biometrics, Ltd., Ramat-Gan, Israel) through a finger probe. A 1-lead electrocardiogram signal was sampled at a frequency of 500 Hz; a reflectance-mode photoplethysmogram (PPG) signal from the right-hand index finger was sampled at a frequency of 500 Hz. Skin conductance (measured in micro-Siemens, μS) was measured with two electrodes that were placed on the volar pads of the distal phalanx in the middle and ring fingers of the right hand; it was sampled at a frequency of 31.25 Hz. The recorded signals were synchronized and processed off-line using MATLAB R2010 scientific software (The MathWorks, Inc., Natick, MA, USA).

#### 2.2.3. Facial Action Coding System (FACS)

The physiological response to pressure stimuli in terms of facial expressions was analyzed using the Facial Action Coding System (FACS). The FACS comprises a list of facial actions (action units (AUs)) that correspond to the movement of specific facial muscles or a group of facial muscles [37]. We used 14 AUs that have been found to serve as valid, reliable, and sensitive indicators of pain [38,39]; they have been utilized in our previous studies in which we also established high inter-rater reliability and agreement [22,33]. The AUs were as follows: Brow lowerer (AU4), cheek raiser (AU6), lid tightened (AU7), nose wrinkler (AU9), upper lip raiser (AU10), lip corner puller (AU12), lip stretcher (AU20), lip presser (AU24), lips part (AU25), jaw dropper (AU26), mouth stretch (AU27), eyelid drop (AU41), eyes closed (AU43), blink (AU45).

#### 2.2.4. Self-Report Rating Scale

Pain ratings following pressure stimuli were acquired using a Pyramid pain scale. This is a graphical rectangular ruler, made out of plastic, 20 cm long and 7 cm wide, on which five colored pyramids of different increasing sizes are drawn on a horizontal base. The region of the scale’s base that has no pyramid above it (the leftward endpoint) indicates no pain = 0. The heights of the pyramids represent the magnitude of pain wherein the highest pyramid (the rightward endpoint) indicates the worst possible pain = 5. This scale was validated in a previous study among both individuals with IDD and cognitively intact peers [33]. Nevertheless, the participants were instructed to rate their pain on a 0-10 numerical rating scale (NRS) as well, which was anchored at 0 = no pain sensation and 10 = worst pain imaginable, which, for participants in the HC group, is more intuitive. 

## 3. Procedures

### 3.1. Training and Pressure Stimulation

The experiment was designed by the “experimental pain” working group of the European Cooperation in the Field of Scientific and Technical Research (COST), termed “Pain assessment in patients with impaired cognition, especially dementia” (TD-1005) of which the authors are members. The aims of this international group are to raise awareness of the subject of pain among individuals with cognitive impairment and to construct a pain assessment tool for this population. The protocol was first tested on healthy volunteers prior to testing individuals with IDD in order to verify the intensity of the pressure stimuli and the ability to endure them for the required duration [33]. 

Prior to actual testing, the participants of both groups underwent a training session in which they were familiarized with the pressure algometer and the PMD-100 device and were trained to use the pyramid scale. During the training session, the participants were administered pressure stimuli of various intensities in the mid-thigh region. The participants were then instructed how to rate their pain using the pyramid and NRS scales. In addition, the subjects were instructed how to sustain their faces in order to capture their facial expressions by the camera.

**Figure 1:**
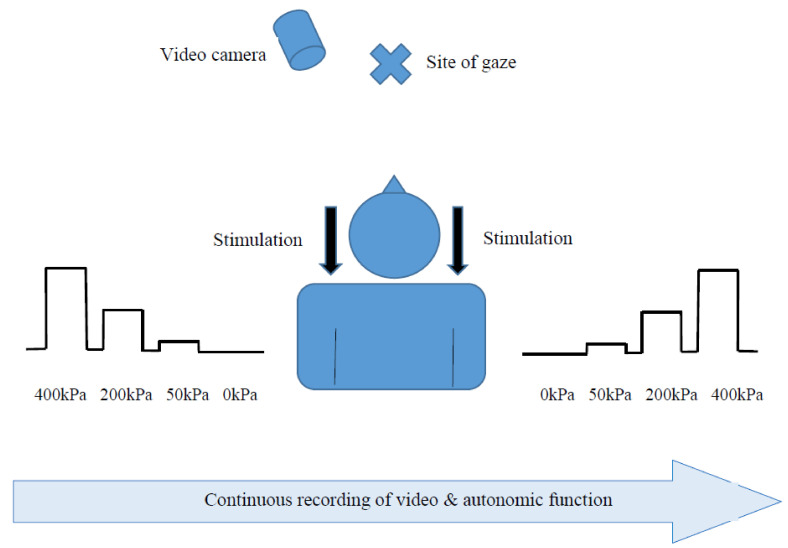
The experimental setup.

After a five-minute break, the experiment began. Figure 1 presents the experimental set-up. The examiner stood behind the subject to administer the stimuli but not to interfere with videotaping. Each subject received a total of six pressure stimuli, administered with the pressure algometer, to the upper part of the trapezius muscle (halfway between the neck line and the shoulder line). The stimuli were administered to the left and right side in an alternate manner (three stimuli on each side). The intensities of the pressure stimuli were 50, 200, and 400 kPa. These stimuli were chosen based on a preliminary experiment conducted on healthy adults in order to evoke one innocuous, one mildly noxious, and one moderately noxious pressure sensation, respectively [33]. The pressure stimuli rose rapidly from a baseline of 0 kPa to the designated magnitude and lasted seven seconds (a two-second increase from baseline and five seconds in the destination intensity). The duration of pressure increase from baseline was similar for all the stimuli so that the evoked facial expressions and physiological responses would be captured at a similar duration for all the stimuli.

The interval between stimuli applied to each body side was two minutes, and the interval between stimuli applied on the same body side was four minutes. These intervals were chosen in order to avoid carry over between stimuli (especially because the stimulation intensities were not randomized) and in order to allow sufficient time for pain rating. The examiner moved the algometer’s probe by about 0.5 cm when returning to a previous location. The rational for lack of randomization in stimulation intensity was based on the preliminary trials. When individuals with IDD received the strongest stimulus before the weaker stimuli due to randomization, they became alarmed and anxious and wanted to withdraw from the experiment. In contrast, when they received the stimuli according to the order of intensity, these individuals could easily endure the entire protocol. 

The participants with IDD as well as controls rated their pain after each stimulus, using the pyramid scale by pointing with their finger to the pyramid that best fit their pain (two participants could not use this scale and instead were asked to report if pain existed or not, and if they said yes, they were asked to report if the pain was mild, moderate, or strong). The participants also provided a number between 0–10 on the NRS. 

### 3.2. Recording and Analysis of the Facial and Bodily Responses

The video camera was recording the entire duration of the protocol. The camera was situated on a tripod 0.5 meter in front of the participant. In order to ensure an optimal position of the face for the purpose of FACS analysis, the participants were asked to look at a green “X” shape that was mounted on the wall in front of them. The behavioral responses of the participants during baseline and pressure stimulation were analyzed retrospectively, frame-by-frame, using the slow-motion option. During baseline, the subjects were instructed not to engage in any specific activity; the analysis of facial expression and freezing was conducted for a random seven-second segment. The analysis of responses during pressure stimulation began at the moment the stimulus was applied, for a duration of seven seconds. The raters observed the video segments of the different conditions (rest, the innocuous, and the two noxious stimuli) in a random order. 

The intensity of most of the FACS’s AUs was coded on a six-point intensity scale ranging from 0 = no action, through 1 = minimal action/trace, to 5 = maximum action [38]. The intensity coding of AU43 was binary; namely, a score of 0 or 5 and the intensity coding of AU45 was on the frequency of blinking. The FACS score for each participant was the sum total of the intensity (or frequency) scores of all 14 AUs. Furthermore, in order to analyze the most pronounced locations of the pain-related AUs, we calculated the frequency of each AU for the two noxious stimuli (200 and 400 kPa). Because, in our previous studies, we found that, in addition to the facial expressions recorded with the FACS, individuals with IDD and CP often responded to the noxious stimuli with body “freezing” or “stillness” [33]; we coded this additional item as well, separately from the FACS. The coding of freezing, which was defined as stillness and/or lack of upper body movement for at least three seconds, was binary (yes/no). 

### 3.3. Analyzing the Physiological Signals

Physiological signals were continuously recorded with PMD-100 throughout the entire protocol duration, and the data for the analysis were extracted off-line. For each of the four study conditions, rest, 50, 200, and 400 kPa, segments lasting 15 seconds, beginning from the application of pressure onto the subjects’ skin, were sampled and averaged [33].

## 4. Data Analysis

Data analysis was conducted with IBM SPSS statistics software (version 25, IBM, New York, USA). The normal distribution was evaluated using the Kolmogorov–Smirnov test. First, the values of the FACS, pain ratings, and autonomic variables were compared between the right and left shoulder. Because there were no body side effects, data from the two shoulders for each variable separately were averaged for use in subsequent analyses. Parametric and non-parametric models with interactions were used to measure group effect (IDD vs. HC) and condition effect (baseline, 50, 200, and 400 kPa) on the following dependent variables: FACS scores, freezing, pyramid scores, NRS scores, HR, HRV, PPGA, and GSR. Post-hoc tests were corrected for multiple comparisons using the Tukey correction. The correlation between each two variables was calculated with Pearson’s or Spearmans’ *r*. The internal consistency of the FACS was assessed using the α-Cronbach test.

Because not all 14 items of the FACS changed similarly during pain within the IDD and HC groups, and in order to learn which FACS items can classify the groups during pain and contribute to pain identification, a two-step cluster analysis was performed on data obtained during the 400 kPa stimulation (the more intense noxious stimulus). Crosstabs analysis and multivariate ANOVA (MANOVA) were then performed to evaluate the association between the clusters and the groups (IDD and HC) and to test group differences (Partial Eta^2^ values assessed the ratio of variance). 

Two-tailed p-values are reported and *p* < 0.05 was considered significant.

## 5. Results

### 5.1. The Study Groups

The IDD group did not differ from the HC group in age (*t*-test: *t* = −1.41, *p* = 0.15) and sex distribution (Mann–Whitney U-test: *Z* = −0.48, *p* = 0.68). Table 1 presents the participants with IDD; it included nine individuals with Down syndrome (DS subgroup; age 33.9 ± 2.35 years) and nine individuals with Unspecified Intellectual Disability (UID subgroup; 39.0 ± 10 years). Individuals with DS and UID did not differ in age (*t* = −1.4, *p* = 0.16), sex distribution (*Z* = −1.82, *p* = 0.12), or their level of IDD (*Z* = −1.89, *p* = 0.07). All but three participants in the IDD group used medications; the medications most frequently used were antihypothyroidism and antipsychotic drugs (Table 1).

### 5.2. Facial Expressions (FACS)

**Figure 2:**
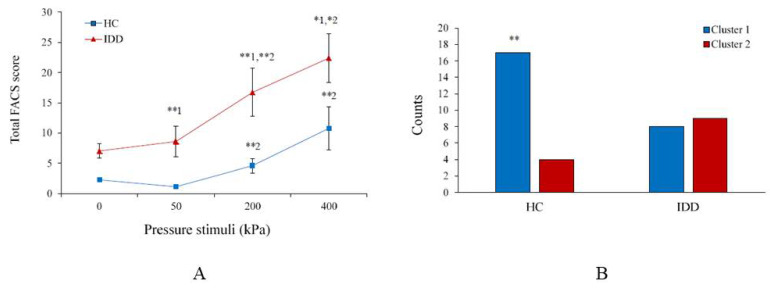
(**A**) The Facial Action Coding System (FACS) scores of individuals with IDD were significantly higher than those of HC regarding all three stimulation intensities (1 = * *p* < 0.05; ** *p* < 0.01), and among both groups the FACS scores increased gradually and significantly from 50 kPa to 200 kPa and from 200 kPa to 400 kPa (2 = * *p* < 0.05; ** *p* < 0.01) (the values denote the group mean ± SEM). (**B**) Clustering for 400 kPa revealed a significant group effect (** *p* < 0.01) in the representation within the two clusters (the values denote the number of participants).

Figure 2A presents the FACS scores in response to pressure stimulation for the IDD and HC groups. Repeated measures ANOVA revealed a significant global effect of group type (*F*(1,34) = 8.76, *p* < 0.01) and of condition (*F*(3,102) = 15.23, *p* < 0.0001) on the FACS scores. Post hoc tests revealed that the FACS scores of the IDD group were significantly higher than those of the HC group over all the conditions (baseline: *t* = −4.15, *p* < 0.0001; 50 kPa: *t* = −8.18, *p* < 0.01; 200 kPa: *t* = −3.0, *p* < 0.01; 400 kPa: *t* = −2.15, *p* < 0.05). Within the IDD group, the FACS scores of individuals with DS were similar to those with UID, except for 200 kPa, in which the scores of the formers were higher (*t* = 2.46, *p* < 0.05). 

The interaction between group and condition was not significant (*F*(3,102) = 1.51, *p* = 0.29), suggesting that the rate of increase in the FACS scores with the increase in stimulation intensity was similar across groups. For both the IDD and HC groups, the overall FACS score of 200 kPa was higher than that of 50 kPa (*t* = −3.1, *p* < 0.01 and *t* = −3.2, *p* < 0.01, respectively), and that of 400 kPa was higher than that of 200 kPa (*t* = −2.4, *p* < 0.05 and *t* = −2.2, *p* < 0.05, respectively). However, this increase in FACS scores was steeper in the IDD group, as indicated by the slopes of the regression line for each group (5.41 vs. 2.82, respectively).

With regard to the individual AUs of the FACS, those that appeared during noxious stimulation, among more than half of the participants within each group, were as follows: for 200 kPa AU7 (11 participants, 61.1%) and AU25 (9, 50%) characterized the IDD group, whereas AU45 (17, 81%) characterized the HC group. For 400 kPa, AU6 (9, 50%), AU7 (11, 61.1%), AU10 (9, 50%), AU25 (10, 55.5%), and AU43 (9, 50%) characterized the IDD group, whereas AU45 (18, 85.7%) characterized the HC group. Table 2 presents the mean frequency of each AU for the innocuous (50 kPa) and noxious stimuli (200 and 400 kPa) among the IDD and HC groups. The table shows that the mean score of several AUs differed between the innocuous and noxious stimulation among the IDD group: AU6, 7, 9, 10, 12, 25, and 43, whereas fewer of them differed between the two noxious stimuli: AU6, 7, 12, and 25 (it significantly increased in the frequency of appearance from 200 to 400 kPa). In contrast, among the HC group, AU41 and AU45 differed regarding the innocuous and noxious stimulation, whereas many more AUs differed regarding the two noxious stimuli (AUs 4, 7, 10, 12, 20, 41, and 43). Table 2 also shows that the IDD group, in contrast with the HC group, exhibited an increased frequency of appearance of most AUs, within each stimulation intensity, but with a reduced frequency of appearance of AU45. 

Cluster analysis for 400 kPa revealed two clusters (ratio = 2.25, average silhouette = 0.6, suggesting good quality). Cluster 1 was characterized by lower AU frequency values, compared with cluster 2. AUs 6, 7, 10, 12, and 25 had the highest predictor importance in the clustering. Figure 2B shows the cluster number crosstabulation. MANOVA revealed a significant effect (*F*(14) = 3.46, *p* < 0.01), suggesting that the groups were significantly different. Most of the participants in the HC group (81%) were classified as cluster 1, whereas the participants in the IDD group were divided between the two clusters: 44.4% and 50% classified as cluster 1 and 2, respectively (one subject was not classified due to missing values). Participants with IDD in cluster 1 did not differ from those with IDD in cluster 2, in age (*t* = −0.04, *p* = 0.96), sex distribution (*Z* = 0.0001, *p* = 1), or IDD diagnosis (*Z* = -0.45, *p* = 0.73), and in none of the self-report and autonomic variables that are described below. The contribution of each AU to the variability between the clusters and the significance of this contribution is ranked by the Partial Eta^2^ that appears in Table 2 (rightward column). AU 45 explained about 35% of the variability between the clusters and AUs 6, 7, and 25 explained about 10, 13, and 10%, respectively, of the variability.

### 5.3. Body Freezing

**Figure 3:**
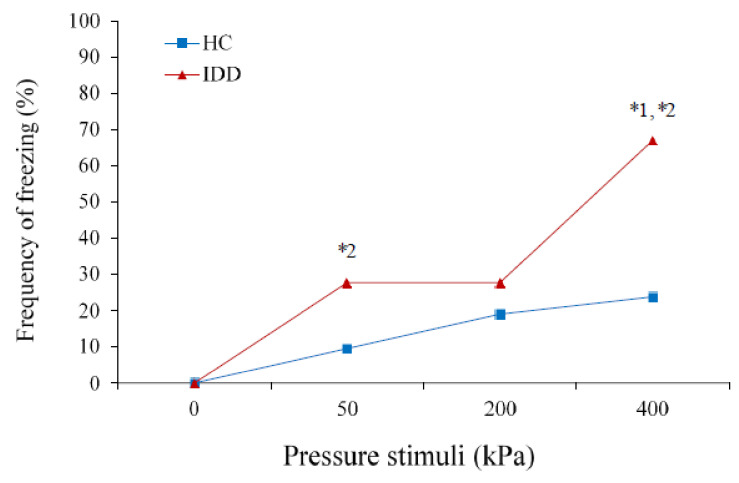
The frequency of freezing among individuals with IDD was significantly higher than that of HC in the most painful stimulus (1 = * *p* < 0.05), and only among individuals with IDD did the frequency of freezing increase significantly from baseline to 50 kPa and from 200 kPa to 400 kPa (2 = * *p* < 0.05) (the values denote the percentages).

Generalized Estimating Equations revealed a significant global effect of group type (χ^2^(1) = 7.31, *p* < 0.05) and of condition (χ^2^(3) = 2.60, *p* < 0.001) on the frequency of body freezing; however, the interaction group X condition did not reach significance (χ^2^(3) = 1.27, *p* = 0.06). Figure 3 presents the frequency (in %) of freezing in response to pressure stimulation among each group.

Post-hoc tests revealed that the frequency of freezing was significantly higher among the IDD than HC group only at 400 kPa intensity (Mann–Whitney U-test: *Z* = −2.65, *p* < 0.05). Among the IDD group, the frequency of freezing significantly increased from baseline to 50 kPa (Wilcoxon signed ranks: *Z* = −2.23, *p* < 0.05), remained the same for 200 kPa, and then significantly increased from 200 to 400 kPa (*Z* = −2.45, *p* < 0.05). Among the HC group, there were no significant increases in the frequency of freezing between the conditions.

Within the IDD group, the frequency of freezing among individuals with DS was similar to those with UID. 

### 5.4. Self-Ratings

**Figure 4:**
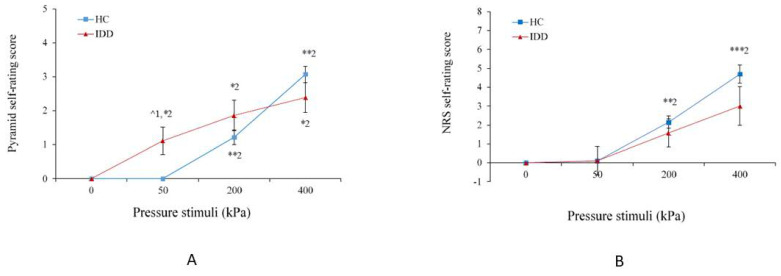
(**A**) The pyramid score for the innocuous 50 kPa stimulus was higher among the IDD group than the HC because the HC group did not rate it as painful (1 = * *p* < 0.05). Among both groups, the pyramid scores increased gradually and significantly from 50 kPa to 200 kPa and from 200 kPa to 400 kPa (2 = * *p* < 0.05; ** *p* < 0.01). (**B**) The numerical rating scale (NRS) scores increased gradually with the increase in stimulation intensity only among the HC group, from 50 kPa to 200 kPa and from 200 kPa to 400 kPa (2 = ** *p* < 0.01; *** *p* < 0.001). The values denote the group mean ± SEM.

Figure 4A presents the median pyramid scores following pressure stimulation in the IDD and HC groups. Generalized Estimating Equations revealed a significant global effect of group type (χ^2^(1) = 158.71, *p* < 0.001) and of condition (χ^2^(2) = 957.39, *p* < 0.001). The interaction group X condition was significant (χ^2^(1) = 6.84, *p* < 0.01). Figure 4A shows that only IDD participants considered the 50 kPa stimulus as painful (Wilcoxon signed ranks: *Z* = −2.3, *p* < 0.05 compared to baseline; Mann–Whitney U-test: *Z* = −2.5, *p* < 0.05 compared to HC). The pyramid scores for 200 and 400 kPa were not significantly different between the IDD and HC groups. Among both the IDD and HC groups, the pyramid scores increased gradually and significantly with the increase in stimulation intensity from 50 kPa to 200 kPa (*Z* = −1.9, *p* < 0.05 and *Z* = −3.16, *p* < 0.01, respectively) and from 200 kPa to 400 kPa (*Z* = −2.2, *p* < 0.05 and *Z* = −3.26, *p* < 0.01, respectively); the increase was steeper in the HC group, as indicated by the slopes (0.79 vs. 1.04, respectively).

Figure 4B presents the average NRS scores following pressure stimulation in the IDD and HC groups. Repeated measures ANOVA revealed no group effect (*F*(1,32) = 0.29, *p* = 0.59), but there was a significant effect of condition (*F*(3,96) = 37.29, *p* < 0.0001) and a significant interaction between group and condition (*F*(3,96) = 3.52, *p* < 0.05). Post hoc tests show that the NRS scores of the HC group gradually increased as stimulation intensity increased: from 50 kPa to 200 kPa (*t*-test: *t* = −6.9, *p* < 0.0001) and from 200 kPa to 400 kPa (*t* = −6.8, *p* < 0.0001), whereas the scores of the IDD group did not significantly change from 50 kPa to 200 kPa (*t* = −1.6, *p* = 0.12) or from 200 kPa to 400 kPa (*t* = −1.8, *p* = 0.08). As with the pyramid scores, the increase was steeper in the HC group, as indicated by the slopes (1.04 vs. 1.62, respectively). 

Within the IDD group, both the pyramid and NRS scores of individuals with DS were similar to those with UID.

### 5.5. Autonomic Variables

**Figure 5:**
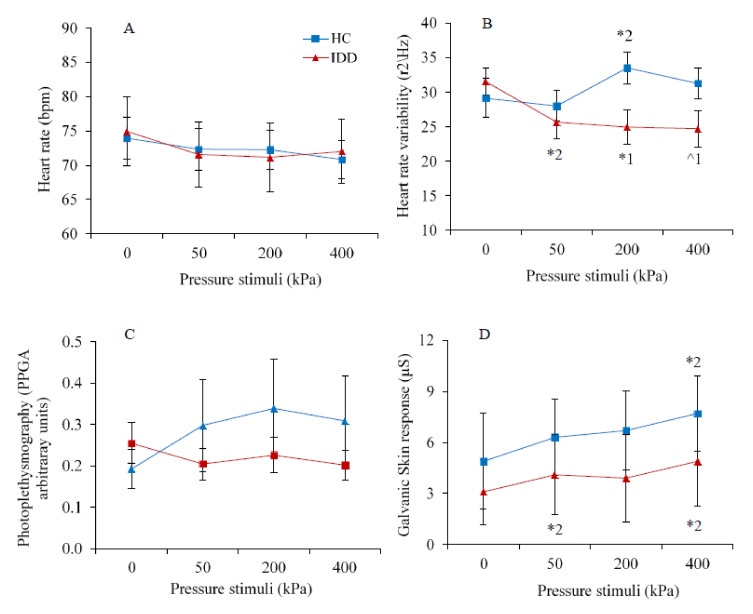
(**A**) The heart rate was not affected by group or stimulation intensity. (**B**) The heart rate variability of individuals with IDD was lower than that of the HC group in the noxious range (1 = * *p* < 0.05, ^ *p* = 0.06) and it increased during stimulation compared to baseline (2 = * *p* < 0.05). (**C**) The photoplethysmograph wave amplitude was not affected by group or stimulation intensity. (**D**) The galvanic skin response among the IDD group increased during stimulation relative to baseline, and among both groups it increased from 200 to 400 kPa (2 = * *p* < 0.05). The values denote the group mean ± SEM.

Figure 5A–D present the average autonomic scores in response to pressure stimulation in the IDD and HC groups.

Repeated measures ANOVA revealed lack of group effect on any of the autonomic variables; however, a significant effect of condition was found on GSR (*F*(3,78) = 5.44, *p* < 0.01). The interaction group × condition was significant for HRV (*F*(3,78) = 2.861, *p* < 0.05) and PPGA (*F*(3,78) = 4.255, *p* < 0.01). Post-hoc tests revealed trends that did not reach significance; HR remained stable across stimulation conditions among both groups (Figure 5A), HRV slightly increased during noxious stimulation only among the HC group but remained stable among the IDD group (Figure 5B), PPGA slightly increased during noxious stimulation only among the IDD group but remained stable in the HC group (Figure 5C), and GSR slightly increased only among the HC group (Figure 5D). 

Within the IDD group, the autonomic variables of individuals with DS were not different from those with UID.

### 5.6. Correlations between Variables

Table 3 presents the correlations between the study variables. Among both the IDD and HC groups, the stimulation intensity correlated significantly only with the FACS scores and self-reports, but with none of the autonomic variables. Furthermore, the FACS scores significantly correlated with self-reports among both groups; therefore, participants who received higher FACS scores also reported higher pain intensity. Among the IDD group only, the FACS scores correlated with GSR; therefore, the higher the FACS scores, the higher the GSR and pyramid scores correlated inversely with HR and PPGA, and the higher the pyramid scores, the lower the HR and PPGA.

## 6. Discussion

The aim was to determine whether, and which behavioral and autonomic indices can differentiate between painful and non-painful conditions and can quantify pain magnitude among individuals with IDD. Facial expressions and pyramid scores fulfilled these two requirements among both the IDD and HC groups. Moreover, compared with the HC group, the facial expressions of the IDD group were enhanced across all stimulation intensities and HRV was slightly lower, suggesting a heightened overall pain responsiveness. 

### 6.1. Indices That Differentiate between Painful and Non-Painful States

In the present study, the facial expressions and self-reports could differentiate between the non-painful stimulation of 50 kPa and those of 200 and 400 kPa among both groups. Among the IDD group, only the self-reports on the pyramid scale could differentiate between the stimuli whereas, among the HC group, both the pyramid and the NRS scores could. There are only a few studies in which the calibrated experimental stimuli were presented to individuals with IDD, and not all of them included painful stimuli. 

In two studies, Symons et al. administered innocuous light touch, deep pressure, cool and warm stimuli (5 s each), and pin pricks (1 s) to individuals with moderate-profound IDD. Neither the FACS scores [28] nor the bodily responses that were scored with the Pain and Discomfort Scale [29] could discriminate between the stimuli. However, the pin prick test was considered mildly painful and was of short duration; perhaps it was insufficiently strong to induce a pronounced response. Barney et al. (2015) administered two mechanical stimuli: light cotton stroking and repeated application of a Von Frey monofilament (temporal summation of pain) to children with IDD [24]. Facial and body gestures analyzed with Batten’s Observational Pain Scale could not differentiate between the two stimuli; however, the minimal responses of the children’s siblings to these two stimuli may suggest that they both were quite mild. Interestingly, repeated Von Frey stimulation did induce a greater increase than did cotton stroking in children’s facial temperature measured with infrared thermography (IRT). Since the autonomic variables measured in the present study failed to differentiate between the painful and non-painful stimuli, the potential use of the IRT would be interesting to explore. More recently, Barney et al. (2020) administered six experimental stimuli: deep pressure, repeated mechanical, light touch, pin prick, cold, and heat to children with cerebral palsy and observed varied levels of facial and bodily responses both among and between the children [31]. However, the authors were unable to determine with certainty whether any of the children had experienced pain.

The inclusion of a control group of cognitively intact subjects in the present study allowed us to determine that the 200 and 400 kPa stimuli were indeed painful and differed significantly in intensity from the innocuous 50 kPa stimulus. The present results corroborate a previous study from our laboratory in which the FACS and pyramid scores of individuals with cerebral palsy and IDD increased significantly from the innocuous to the noxious pressure [33]. Note that, although the NRS’s and the pyramid scale’s ratings correlated with one another and with the stimulation intensity among both groups, the use of NRS among the individuals with IDD was generally ineffective; their ratings increased but not sufficiently enough to differentiate the noxious from the innocuous stimuli. Accordingly, their facial expressions did not correlate with their NRS scores, only with their pyramid scores. Thus, we concluded that the FACS and the pyramid ratings are valid indicators for identifying a painful condition among individuals with mild-moderate IDD due to Down syndrome or an unspecified origin, as herein, and due to cerebral palsy, as in our previous publication.

Specifically, seven AUs could differentiate between the innocuous and noxious stimuli among the IDD group: cheek raiser (AU6), lid tightened (AU7), nose wrinkle (AU9), upper lip raiser (AU10), lip puller (AU12), lips part (AU25), and eyes closed (AU43), which, combined, suggest an elevation of the cheek area along with mouth opening. AUs 4, 6, 7, and 25 were also reported by Symons et al. (2010) as the most prominent ones following the experimental stimuli [28], and AUs 9,10, and 12 were reported to frequently occur during painful clinical situations e.g., immunization and dental treatment, e.g., [19,20,25,40]. Interestingly, AUs 6, 7, 9, 10, and 25 also comprised the “face of pain” of elderly people with dementia [41]. These data combined may suggest that the seven aforementioned AUs may be sufficient to identify pain among non-verbal individuals with IDD. Notably, altogether, the HC group responded differently during the transition from innocuous to noxious stimulation and exhibited a significant increase only in eyelid drop (AU41) and blink (AU45), suggesting that facial expressions among individuals with mild-moderate IDD are unique in this respect. 

It should be pointed out that, despite the inability of the HR, HRV, PPG, and GSR to discriminate between noxious and innocuous stimuli in either group, we cannot rule out the possibility that the noxious stimuli may have been insufficiently extended or strong in order to produce the expected effect. Previous studies measured changes in heart rate, electro-dermal activity, oxygen saturation, and salivary amylase among children and adults with IDD during painful clinical conditions including invasive venipuncture and bronchial tube exchange, and post-operatively, e.g., [25,26,27,42]. However, others reported a lack of or negligible changes in, for example, the heart rate in these conditions [18,42]. Such conditions are obviously more vigorous than the present noxious mechanical stimuli. Nevertheless, the changes in autonomic function in the aforementioned studies did not necessarily correlate with the proxy impression of the subjects’ pain or with the observed behavior, suggesting that it may reflect aspects other than pain per se. Thus, although autonomic variables may be affected by acute clinical pain conditions and are easy to monitor; their use as pain indicators requires further investigation with stronger experimental stimuli than those used here, and perhaps with additional stimulation modalities. A new approach to analyze the electrodermal activity may hold promise pending further examination [43].

### 6.2. Indices That Can Differentiate between the Intensities of Noxious Stimuli 

Another aim of the present study was to investigate which indices can differentiate between the two noxious stimuli and quantify their magnitude among individuals with IDD. Both the FACS and pyramid scores correlated with the stimulation intensity and significantly increased from 200 to 400 kPa, suggesting that they can encode not only the presence of pain, but also the intensity of mild vs. moderate pain. These findings, which corroborate our previous study among individuals with cerebral palsy [33], are clinically important because the degree of facial expressions (and the pyramid scores for those who can provide them) can be used to detect changes in pain intensity while following up the worsening of pathological conditions or, alternatively, pain management programs. This is particularly important, given that the subjective indicators used in the present study, namely, the autonomic variables, did not correlate with the stimulation intensity, with one exception. GSR exhibited a significant increase from 200 to 400 kPa among both groups. Thus, monitoring GSR in addition to FACS (and self-ratings when possible) may increase the accuracy of pain intensity evaluation. 

Here again, almost no overlap occurred between the groups regarding the specific AUs that could differentiate between the noxious stimuli. Among the IDD group, four AUs were identified: cheek raiser (AU6), lid tightened (AU7), lip corner puller (AU12), and lips part (AU25), which increased in their magnitude of appearance. The partial Eta^2^ values indeed corresponded with AU6, AU7, and AU25 as being the major contributors to the facial expression of moderate pain among the IDD group. Lid tightened and lip puller were the only AUs that overlapped with the HC group, who exhibited, in addition, increases in brow lowerer, lip raiser, lip stretcher, eyelid drop, and eyes closed; namely, the HC group presented more diverse facial actions than the IDD group. We could not find additional studies in which various intensities of calibrated noxious stimuli were administered to individuals with IDD. 

Interestingly, although the frequency of body freezing could not differentiate between noxious and innocuous stimuli, it could differentiate between the two noxious stimuli; it characterized the majority of the IDD group at 400 kPa. Body freezing or stillness has been observed among individuals with IDD, both following clinical painful insults [22,44] and experimental stimuli [28,29,33], and it can dominate despite the pronounced facial expressions. This dissonance may mislead care givers because individuals subjected to freezing may appear indifferent to pain, but in essence, they are significantly affected by it, as the FACS and self-ratings here showed. Thus, body freezing as a possible atypical index of acute pain can be incorporated during pain assessment, but because it is coded in a binary fashion, it cannot be a stand-alone indicator of pain intensity, rather, only a confirmation indicator.

### 6.3. Comparisons between Individuals with IDD and HC

Three indices were found to differentiate between the IDD and HC groups: the FACS scores of the IDD group were increased, the body freezing was more frequent, and the HRV was lower in the IDD group. Considering that reduction in HRV is typical of stressful/painful conditions [45,46], the three findings suggest that individuals with IDD due to Down syndrome or to a condition of unspecified origin present enhanced behavioral and physiological responses to noxious stimuli. This conclusion is supported by the increased behavioral [33] and pain-related brain potentials [34] found following calibrated noxious stimuli among individuals with IDD due to cerebral palsy. One exception was that the IDD group also exhibited an increased pulse rate and GSR compared with controls, both at baseline and during stimulation, whereas the participants here had a reduced HRV, compared with controls during stimulation only. These variations in the autonomic responsivity among the aforementioned IDD groups may stem from the IDD etiology; nevertheless, they suggest an overall increased reactivity in IDD. Enhanced behavioral responses to mildly painful calibrated mechanical stimuli were also reported among children with IDD, compared with their siblings [24] and compared with control children [30]. 

The increased pain responsivity of individuals with IDD corresponds to a lower pain threshold measured among individuals with Down syndrome, cerebral palsy, or unspecified IDD [35,36,47,48]; although, see [49]. Thus, the enhanced pain behavior in IDD may result from increased sensitivity to noxious stimuli. Alternatively, but this does not conflicting with the aforementioned, there is the possibility that the increased pain reactivity results from reduced modulatory control. Recent imaging studies revealed an increased activation of the somatosensory cortex of children with IDD, compared with control children during venipuncture [50] as well as a reduced activation of the prefrontal cortex among individuals with Down syndrome compared with controls [51,52], in addition to decreased volume in the brain stem, among other structures [53]. Considering that the frontal cortex and the brain stem are highly involved in pain modulation [54,55], individuals with IDD may lack sufficient control over nociceptive input and its interpretation, rendering them more sensitive and reactive to noxious stimuli. 

The cluster analysis performed for the facial action units recorded during 400 kPa stimulus (the higher noxious stimulus) revealed, however, that pain behavior within the IDD group was not uniform. Specifically, about half of the participants responded similarly to the HC group (cluster 1) and the other half responded more vigorously (cluster 2). Except for somewhat higher pyramid scores among participants in cluster 2, the two IDD clusters were similar in the remaining tested variables. Despite the within-group variability, the objective and semi-objective indices suggest that individuals with IDD are more responsive to pain than are the cognitively intact individuals; therefore, any suspected pain condition or any complaint should be carefully monitored and evaluated. 

An interesting discrepancy was observed between the semi-objective and the subjective pain indicators within the IDD group; the increase in the FACS scores (and in freezing) with stimulation intensity was steeper than that of controls. However, the increase in self-reports was milder than in the controls. This discrepancy may mean that, although self-reports are obtainable from individuals with mild-moderate IDD, they may underestimate the pain experienced by these individuals. Individuals with IDD may not perceive the subtleties of the self-report scales to the extent that they can adequately quantify the changes in their perceived pain. The stimulus-response relationship for the pyramid scores, but not for the NRS scores, emphasizes the advantage of graphical scales, which may be more intuitive to use. The pyramids symbolize pain intensity by their height and their gradual increase, which enable a person to grasp the concept of magnitude in a more intuitive manner. Cube and box scales have also enabled proper self-reports from individuals with IDD [56,57]; however, controversies exist with regard to the use of face scales and colored visual analog scales [19,22,25,48,57,58,59,60]. Thus, although self-reporting using graphical scales is definitely a valid means among individuals with IDD who can communicate their experiences, underestimation should be considered and their combination with observational measures is preferable [61,62].

### 6.4. Limitations 

There are several limitations to consider. First, the results apply to individuals with mild-moderate IDD. Thus, the ability of the identified AUs or other pain indices to detect and quantify pain should also be validated among individuals with more severe IDD. Second, several AUs may also indicate distress; therefore, the recorded FACS responses among the individuals with IDD in particular may reflect a combination of pain and distress. Third, the mild and moderate stimulation intensities noted here may not mimic painful clinical conditions; however, they were chosen both for validation and because of ethical considerations.

### 6.5. Conclusions and Impact

To the best of our knowledge, this is the first study in which pain responses to calibrated noxious stimuli are recorded among adults with Down syndrome or unspecified IDD, compared to cognitively intact peers. The results suggest the following conclusions: (1) Individuals with IDD may perceive noxious stimuli as more painful than normal. (2) FACS scores and self-reports using the pyramid scores can consistently differentiate between non-painful and painful conditions, and between mild and moderate pain. The previously established high inter-rater reliability and agreement of the FACS [22,33] and the validity and sensitivity of the FACS and the pyramid scale, found herein and in our previous study [33], suggest that the psychometric properties of these tools among individual with IDD due to CP, Down syndrome, and unspecified origins are good. (3) Increased facial reactions can co-exist with body freezing among individuals with IDD. 

Taken together, the increased (and at times atypical) pain responses among individuals with IDD, along with the health hazards that they may be exposed to, put them at risk of experiencing intense pain throughout their lifetime. The increased and greater pain responses of individuals with IDD necessitate meticulous monitoring of any possible sign of distress/pain and the administration of pain alleviation medication accordingly. Care givers face great challenges in their attempt to provide appropriate care for individuals with IDD, and often rely on pain behavior [63]. Although experimental pain may not necessarily mimic acute and chronic pain responses in clinical settings, the present results are encouraging in that they provide evidence that self-reports using appropriate means is plausible and valid, and that a combination of specific facial expressions may help detect and quantify pain among those who cannot self-report. Because each pain measure contributes unique information about an individual, the combined use of both measures is beneficial and recommended.

## Figures and Tables

**Table 1:** Characteristics of individuals with intellectual and developmental disabilities (IDD).

Subject	Sex	Etiology	Age	ID Level	Medications
1	F	DS	37	Mild-moderate	Antihypothyroidism
2	F	DS	34	Mild	None
3	F	DS	31	Moderate	Muscle relaxants, steroids
4	F	DS	31	Moderate	Antipsychotic, muscle relaxants, steroids
5	M	DS	32	Moderate	Antihypothyroidism, antipsychotic
5	M	DS	36	Mild	Antihypothyroidism, antihypertention
7	F	DS	33	Mild-moderate	Muscle relaxants, antihypertention
8	F	DS	37	Mild-moderate	Antihypothyroidism, antihypertention
9	F	DS	34	Mild-moderate	Antihypothyroidism
10	M	UID	49	Mild	Antipsychotic, muscle relaxants
11	M	40	UID	Mild	None
12	M	52	UID	Mild-moderate	Antihyperthyroidism
13	M	UID	38	Mild-moderate	None
14	F	UID	26	Mild-moderate	Antipsychotic
15	M	UID	40	Mild	Antidepressant
16	M	UID	27	Mild	Antidepressant
17	F	UID	50	Moderate	Antihypertention
18	F	UID	29	Mild	Antiepileptic, antihypertension Antihypothyroidism, muscle relaxants

DS = Down syndrome, UID = unspecified intellectual disability, M = male, F = female.

**Table 2:** Mean frequency scores (SD) of each FACS action unit during innocuous and noxious stimulation.

	50 kPa		200 kPa		400 kPa		
	IDD	HC	IDD	HC	IDD	HC	Partial Eta^2^
Brow lowerer (AU4)	0.83 (1.5) *	0.0 (0)	1.11 (1.7)	0.57 (1.3)^#^	1.29 (1.9)	1.14 (1.9)	0.002
Cheek raiser (AU6)	0.61 (1.4) # *	0.0 (0)	1.56 (2.2) # **	0.05 (0.2)	2.06 (2.3)	0.86 (1.5)	0.096 *
Lid tightened (AU7)	0.89 (1.7) # *	0.0 (0)	2.11 (2.2) # **	0.43 (1.0) #	2.88 (2.4) *	1.24 (2.0)	0.131 *
Nose wrinkle (AU9)	0.28 (0.9) #	0.0 (0)	1.22 (2.1) *	0.0 (0)	1.24 (2.0)	0.52 (1.4)	0.046
Upper lip raiser (AU10)	0.39 (1.2) #	0.0 (0)	1.33 (2.0) **	0.05(0.2) #	1.88 (2.2)	0.90 (1.8)	0.062
Lip corner puller (AU12)	0.44 (1.3) #	0.0 (0)	1.50 (2.1) # **	0.0(0) #	2.12 (2.4)	1.14 (2.1)	0.049
Lip stretcher (AU20)	0.44 (1.3)	0.0 (0)	1.00 (1.5) **	0.05 (0.2) #	0.94 (1.6)	1.14 (1.9)	0.0030
Lip pressor (AU24)	0.44 (1.4)	0.05 (0.2)	0.56 (1.3)	0.10 (0.3)	0.59 (1.3)	0.71 (1.6)	0.002
Lips part (AU25)	1.00 (1.5) # *	0.0 (0)	1.44 (1.8) # **	0.0 (0)	1.88 (1.9) *	0.71 (1.9)	0.095 *
Jaw dropper (AU26)	0.72 (1.0 **	0.0 (0)	0.89 (1.4) **	0.0 (0)	1.35 (1.9)	0.57 (1.5)	0.053
Mouth stretch (AU27)	0.29 (0.6) *	0.0 (0)	0.72 (1.5) *	0.0 (0)	0.94 (1.7)	0.29 (1.1)	0.0540
Eyelid drop (AU41)	0.72 (1.2) *	0.05 (0.2) #	0.89 (1.4)	1.05 (1.8) #	1.06 (1.8)	1.86 (2.3)	0.037
Eyes closed (AU43)	0.33 (1.2) #	0.05 (0.2)	1.39 (2.1)	0.38 (1.1) #	1.82 (2.1)	1.52 (2.2)	0.005
Blink (AU45)	1.22 (1.3)	1.00 (0.9) #	1.00 (1.6)	1.95 (1.6)	0.35 (0.8) ***	2.43 (1.8)	0.346 ***

IDD = intellectual and developmental disability, HC = cognitively intact healthy controls, FACS = facial action coding system, AU = action unit. # denotes significant within-group differences between 50 and 200 kPa (in the 50 kPa column) and between 200 and 400 kPa (in the 200 kPa column) (# *p* < 0.05, 2-tailed *t*-test). Asterisks in the IDD columns denote significant between-group differences for each stimulation intensity (* *p* < 0.05, ** *p* < 0.01, *** *p* < 0.001, 2-tailed *t*-test). Asterisks in the Eta^2^ column denote the significance of the contribution to the clustering for the 400 kPa stimulus (* *p* < 0.05, *** *p* < 0.0001).

**Table 3:** Correlation coefficients between the study variables.

		FACS	Pyramid Scale	NRS	HR	HRV	PPGA	GSR
Simulation intensity	ID	0.41 **	0.57 ***	0.38 **	−0.05	−0.24	0.11	0.23
	HC	0.41 **	0.89 ***	0.82 ***	−0.85	0.13	−0.76	0.19
FACS	ID		0.37 **	0.06	0.26	0.05	0.14	0.31 *
	HC		0.44 **	0.49 ***	−0.09	0.05	−0.09	−0.05
Pyramid scale	ID			0.68 ***	−0.31 *	−0.15	−0.37 **	−0.04
	HC			0.94 ***	−0.13	0.05	−0.04	0.21
NRS	ID				−0.18	0.02	−0.16	−0.05
	HC				−0.02	−0.01	0.07	0.16
HR	ID					−0.27 *	0.07	0.33 *
	HC					−0.35 **	0.34 **	0.27 *
HRV	ID						−0.18	−0.17
	HC						−0.37 **	−0.05
PPGA	ID							0.39 **
	HC							−0.02

Coefficients are of Pearson’s or Spearman’s tests: * *p* < 0.05, ** *p* < 0.01, *** *p* < 0.01; ID = intellectual disability, HC = healthy controls, FACS = facial action coding system, NRS = numerical rating scale, HR = heart rate, HRV = heart rate variability, PPGA = photoplethysmograph wave amplitude, GSR = galvanic skin response.

## Data Availability

Restrictions apply to the availability of these data. Data was obtained after permission from the legal guardians and are available from the authors with the permission of the institutional review board.
